# Mobile Apps for Foot Measurement in Pedorthic Practice: Scoping Review

**DOI:** 10.2196/24202

**Published:** 2021-03-04

**Authors:** Muhammad Ashad Kabir, Sheikh Sowmen Rahman, Mohammad Mainul Islam, Sayed Ahmed, Craig Laird

**Affiliations:** 1 School of Computing and Mathematics Charles Sturt University Bathurst, NSW Australia; 2 Department of Computer Science and Engineering Chittagong University of Engineering and Technology Chittagong Bangladesh; 3 Verizon Media Sunnyvale, CA United States; 4 School of Health and Human Sciences Southern Cross University Queensland Australia; 5 Foot Balance Technology Pty Ltd Westmead, NSW Australia; 6 Pedi Wiz Digital Technology Westmead, NSW Australia; 7 EYM (Ease Your Motion) Pty Ltd Westmead, NSW Australia; 8 Walk Easy Pedorthics Tamworth, NSW Australia

**Keywords:** foot measurement, foot scanning, mobile app, custom shoes making, apps review, diabetic foot, pedorthics, footcare

## Abstract

**Background:**

As the use of smartphones increases globally across various fields of research and technology, significant contributions to the sectors related to health, specifically foot health, can be observed. Numerous smartphone apps are now being used for providing accurate information about various foot-related properties. Corresponding to this abundance of foot scanning and measuring apps available in app stores, there is a need for evaluating these apps, as limited information regarding their evidence-based quality is available.

**Objective:**

The aim of this review was to assess the measurement techniques and essential software quality characteristics of mobile foot measurement apps, and to determine their potential as commercial tools used by foot care health professionals, to assist in measuring feet for custom shoes, and for individuals to enhance their awareness of foot health and hygiene to ultimately prevent foot-related problems.

**Methods:**

An electronic search across Android and iOS app stores was performed between July and August 2020 to identify apps related to foot measurement and general foot health. The selected apps were rated by three independent raters, and all discrepancies were resolved by discussion among raters and other investigators. Based on previous work on app rating tools, a modified rating scale tool was devised to rate the selected apps. The internal consistency of the rating tool was tested with a group of three people who rated the selected apps over 2-3 weeks. This scale was then used to produce evaluation scores for the selected foot measurement apps and to assess the interrater reliability.

**Results:**

Evaluation inferences showed that all apps failed to meet even half of the measurement-specific criteria required for the proper manufacturing of custom-made footwear. Only 23% (6/26) of the apps reportedly used external scanners or advanced algorithms to reconstruct 3D models of a user’s foot that could possibly be used for ordering custom-made footwear (shoes, insoles/orthoses), and medical casts to fit irregular foot sizes and shapes. The apps had varying levels of performance and usability, although the overall measurement functionality was subpar with a mean of 1.93 out of 5. Apps linked to online shops and stores (shoe recommendation) were assessed to be more usable than other apps but lacked some features (eg, custom shoe sizes and shapes). Overall, the current apps available for foot measurement do not follow any specific guidelines for measurement purposes.

**Conclusions:**

Most commercial apps currently available in app stores are not viable for use as tools in assisting foot care health professionals or individuals to measure their feet for custom-made footwear. Current apps lack software quality characteristics and need significant improvements to facilitate proper measurement, enhance awareness of foot health, and induce motivation to prevent and cure foot-related problems. Guidelines similar to the essential criteria items introduced in this study need to be developed for future apps aimed at foot measurement for custom-made or individually fitted footwear and to create awareness of foot health.

## Introduction

### Background

Poor foot health is often linked to poor performance in both personal and work life [1]. Various anatomical and biochemical factors are responsible for the deterioration of foot health, along with overuse, injury, and external trauma. Maintenance of foot health is necessary to keep humans mobile and independent, and consequential negligence can often cause psychological strain along with physical pain [2].

In an effort to combat problems related to foot health, clinical treatment programs have been widely adopted [3,4]. These programs consist of clinical interventions, and most of the time require clinicians and patients to have regular face-to-face contact for over 1 year. Such interventions have shown variable efficacies due to fluctuations in adherence by patients over time [5-7]. These rigorous health programs can sometimes be time-, resource-, and cost-intensive, and can also be inconvenient for patients given that foot problems increase the possibility of impeding movement capabilities [8,9]. Accordingly, there is a need for novel, low-cost, and widely accessible tools to accurately scan and measure patients’ feet, and provide health feedback to patients. This has become a necessity as many patients face significant barriers related to achieving clinical treatments.

The advancement and accessibility of mobile app technology in recent years have enabled efforts to translate the same traditional clinical treatments and intervention programs in the development and growth of the use of foot health mobile apps. The outcome is the development of mobile apps that can provide insight into patients’ feet by leveraging the processing capabilities of mobile sensors such as depth-sensing cameras, multicameras, infrared sensors, and features like augmented reality. Many such apps use algorithms and data-mining techniques to suggest foot- and shoe-related solutions based on the procured foot measurement values, whereas others stop at providing basic information such as the suggested size of a shoe based on foot form and suggested forefoot or toe exercises.

Despite an overall increase in app use for foot health conditions, the analysis of several apps belonging to this category has led to the discovery of various problems related to usability, design, and functionality; the limited availability of free apps; the lack of provision for user consent; and, particularly, the lack of certification and the poor quality of the information displayed to achieve their most important goal (ie, to improve patient outcomes) [10-12]. Consequently, these shortcomings have raised questions about the efficacy and applicability of mobile apps used for foot measurement and scanning [10-12].

### Objective

To the best of our knowledge, no study has extensively explored the current scenario of the commercial mobile app market to review and scientifically evaluate apps related to foot measurement. The abundance and rapid growth of such foot measuring apps in app stores, along with the increased adoption rate of these tools by the public, necessitates an assessment of this rapidly growing market. Therefore, the objective of this scoping review was to evaluate the published foot measurement apps in the two major commercial app stores (Apple App Store and Google Play Store). We evaluated the specific criteria of foot properties and the criteria of software quality characteristics, along with the viability of these apps for use as professional tools for foot measurement by pedorthists, podiatrists, orthotists, and individual users. We also investigated the potential of these apps to increase awareness of foot health and foot-related problems.

## Methods

### App Search Procedure

**Figure 1:**
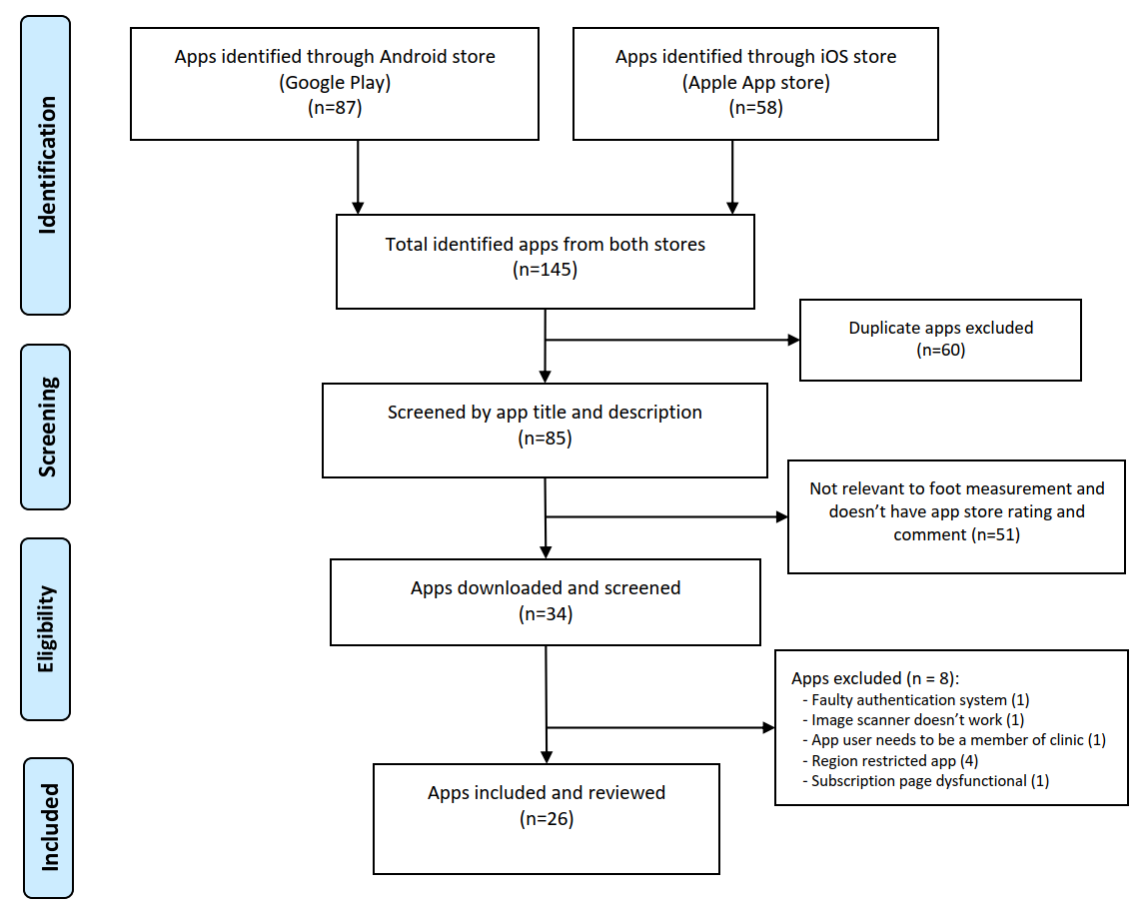
Flow diagram of study methods.

This review included apps found in the official mobile app stores: Apple App Store and Google Play Store. An electronic search was performed between July and August 2020 in the two app stores for iOS and Android mobile devices, respectively. The search process did not consider any subcategories to which the apps belonged in the app stores. The following terms were used to search for foot measurement apps across the app stores: “foot scanner,” “foot app medical,” “foot measure,” “feet,” “measure foot,” and “foot length.” Region-restricted apps were not considered. The methodology used in this study for the identification, screening, and selection of the apps’ matching criteria is displayed in Figure 1.

A secondary search was performed to identify apps that were intended to be used with advanced imaging sensors or other hardware requirements using the following keywords: “foot size,” “shoe size,” “3D foot,” and “foot scan solutions.” The results of this latter search were not limited by language, app store description, or rating. No restrictions were imposed in both searches across the different app stores. The search results obtained using the enlisted terms varied significantly between the app stores. As a result, the exact search using the same terms was performed multiple times across different devices to minimize the variance of app indexing and to construct the final inclusion list of foot measurement apps.

The investigation, screening, and extraction phases of the app search and selection processes were performed by the investigators collaboratively. All investigators involved in this operation contributed equally by maintaining a separate list of apps they found within the app stores using the inclusion and exclusion criteria that were finalized for this procedure (see Measures Used in Apps Selection section below). The investigators acted independently on their own smartphone(s) to determine apps fit for selection. During the merging of the app lists, there were conflicts regarding some apps. One such case was an app encountered by one investigator who rejected it from their list, but the same app was included by another investigator. Such cases of discordance were resolved by mutual discussion among the investigators until consensus was reached. The resulting lists of apps were merged, producing the final list of included apps for analysis.

### Raters

Three expert raters were selected in line with the proven approach described by Stoyanov et al [13], including (1) a software developer with a Master’s degree in software engineering and more than 10 years of mobile app development experience in a renowned company, along with extensive experience using various camera/depth sensors in mobile apps; (2) a computer science graduate with 2 years of mobile app (in particular iOS apps) development experience; and (3) a final-year Bachelor of Computer Science student with 2 years of mobile app development experience. In addition, domain experts (two pedorthists) in the research team trained and educated the raters about the foot measurement processes and the footwear industry.

The raters independently rated all of the apps from the final list of apps compiled by the investigators. Their responses were recorded in a standardized response form (Google Forms) and respective rater response data were extracted from the spreadsheet attached to the form.

### Measures Used in Apps Selection

The criteria for the selection of foot measurement apps were based on whether the app serves a purpose that involves measurement of the user’s foot. An app was selected for inclusion if it met the following criterion: the app was deemed to be a foot measurement app after screening by title, store description, and store rating relevance. An app was excluded from the study if it met one of the following exclusion criteria: (1) it has neither a star rating nor a user comment on the app store listing page; (2) an identical app from the same developer or publisher is available in both app stores (a duplicated app), and therefore it was excluded from one platform; (3) it is inaccessible/unusable due to region restrictions; and (4) it has been reskinned with a new user interface over an existing app, and hence it is deemed to be the same app in terms of functionality. 

### Modification of Existing Health App Rating Tools

#### Selection of Domains and Rating Tool Development

We hypothesized that to properly rate an app with respect to its appropriateness and usability, the app should include multiple features to enable scientifically evaluating its value as a commercially viable foot measurement product. A standardized rating tool would be helpful in this case, especially when there are numerous relevant apps in the app stores. Consequently, an extensive review of prior guidance documents and tools for rating mobile apps was performed to identify the fundamental domains and criteria for determining an app’s usefulness and rating. After reviewing several app rating guidance documents and tools, we concluded that each app has its own uniqueness for particular categories of application. We took into consideration prior studies on software quality assessment, and emphasized the key software quality characteristics such as usability, reliability, functionality, and efficiency [14-17]. Our aim was to further build and extend on prior rating tools such as the Mobile App Rating Scale (MARS) [13], end-user version of the MARS (uMARS) [18], and MARS for financial apps (FinMARS) [19], and adapted these to the required evaluation suite for foot measurement apps. With this in mind, we selected the different categorical domains from each rating tool, and proposed an extended version of the mobile app rating tool for foot measurement (FootMARS). This rating tool consists of modifications that we hypothesized to be more important for a foot measuring app. FootMARS considers individual items that were found during the review of the included foot measure apps relevant to the same goal. The finalized rating tool model with the updated overarching domains and individual category items are illustrated in Table 1.

**Table 1:** Extended foot measurement app rating domains and criteria.

Domain	Criteria
App metadata	App platform App store rating App store description App store URL Number of downloads Origin Developer
App classification	App subcategory Applicable age groups App price
Aesthetics	Layout consistency and readability Content resolution Visual appeal Group targeting according to app content
General app features	Social sharing feature Authentication feature User onboarding interfaces Content customization Visual information Data export options Subscription options
Performance and efficiency	Bootup efficiency Accuracy of features and components Responsiveness of app Frequency of app crash Overheating device issues Battery life impact
Usability	Ease of use Navigational accuracy Gestural design Interactivity and user feedback
Measurement-specific functionality	Measurement of foot length and width Measurement of foot medial arch height Measurement of foot instep or joint girth Measurement of short or long heel girth Measurement of heel width Measurement of shoe size Measurement of forefoot tilt/rotation Additional setup Reconstruction of 3D foot model Additional out of scope features
Transparency	User consent Accuracy of store description Credibility/legitimacy of source Feasibility of achieving goals
Subjective quality	Overall star rating Overall app purchase preference Overall app recommendation Frequency of use based on relevance
Perceived impact of app on users	Awareness induction behavior Knowledge enhancing behavior Scope to improve attitude toward foot health Scope to reduce negligence toward foot Scope to induce foot-related help-seeking behavior

The key domains essential for evaluating foot measurement apps were identified as follows: app classification, aesthetics, general features, performance and efficiency, usability, measurement-specific functionality, transparency, subjective quality, and the app’s perceived impacts on users.

The app quality criteria clustered around the domains, excluding the metadata section, were used to build the app rating scale. Depending on the type of question asked to assess the criteria, each item can score a response as a 5-point Likert scale or a binary response (1 or 5). Cases were found in certain categorical items that were not applicable; thus, the “not applicable” rating was introduced to the scale. Other cases displayed complexity in gaining access to certain types of information, and therefore a rating option “unknown” was added. A few subscale items were excluded from the quantitative measurement since they provided qualitative descriptions about apps that could not be weighed quantitatively. These items are the app metadata domain items, applicable age group item, and app subcategory item.

#### App Metadata

General information about the apps was abstracted as app metadata from the respective app stores under the app classification category. App metadata include information such as app platform, app URL, store rating, store description, number of downloads, developer information, and origin. However, this information has no impact on the rating scale. App metadata were extracted systematically by two investigators from each app store on a Google Sheet and this dataset was cross-verified by a third investigator for data anomalies.

#### App Classification

Through an extensive review of prior work on foot measurement and related technologies [20-23], the apps were subcategorized depending on their type of functionality into the following groups: (1) simple size-unit converter, (2) 2D foot scanner, (3) 3D foot scanner, (4) shoe recommender, (5) foot tilt calculator, and (6) foot progress tracker.

Subcategory 1 (simple size-unit converter) apps are the simplest type of apps that take user input values for foot shape and dimensions to produce another category of size or shape, which can be related to both shoe and foot properties. Subcategory 2 (2D foot scanner) apps are more advanced in comparison as they use imaging sensors to acquire 2D data about foot images and use algorithms to compute the user’s feet dimensions. The 3D scanner apps (subcategory 3) generally require state-of-the-art techniques and external hardware such as 3D imaging sensors or dimensional digitizing devices. This type of app is generally capable of providing an array of user feet dimensions. The shoe recommender (subcategory 4) and foot tilt calculator (subcategory 5) apps are modified versions of 2D and 3D scanners that do not directly output raw measurement information but rather transform this information into more consumer-friendly, useful views, and derived information. Foot progress trackers (subcategory 6) are common apps that were not specifically designed for applications to feet, but this category was nevertheless included in the study because of the ability of these apps to track measurements of different foot areas over time, either manually or using in-built measurement techniques.

#### Aesthetics

The current market is dominated by hundreds of thousands of apps that are competing in similar categories with similar functions and outcomes, but are only preferred based on more visually appealing features. Visual appeal is just as important to the success of a commercial product as its core functionality and performance. A proper layout and organization of an app’s user interface elements can sometimes be the difference between an app’s success and its downfall. This trend is also seen in modern foot scanning apps, which are assessed according to their visual appearance and organization of their layout. Therefore, the key element of a good interface design is to make it clear and simple for users [24]. For measuring the aesthetic properties of an app, the raters considered three factors: (1) the quality of user interface elements in apps, specifically the resolution of images and icons; (2) the overall look and feel of the app’s color sets and theme; and (3) the complexity of the contents of the individual screens in the apps.

#### General Features

Although providing options for as many measurement dimensions as possible is important for a foot measurement app, there are general features that the app must also provide, such as the ability to share and export data to other apps and in various data formats. This feature was selected as a rating item because if it is possible to share data, users are less likely to waste time sharing their foot size information on other platforms, which may be a benefit. An authentication feature is also considered to be a good option. For example, when data are stored against a user’s credentials, the data are likely to be stored in the cloud, thus removing the user’s dependencies on that particular mobile device on which the app is installed. Over recent years, the importance of content customization and the amount of visual information shown in apps have been found to improve user value; therefore, these features were also included for rating. Additionally, if the app provides subscription packages that may affect user experience in any way, these should also be considered. Negative weights were calculated for rating metrics if the apps had any of the following: faulty authentication system, faulty subscription pages, region restrictions, and technical issues in image scanning, as crucial app features. All of these points were considered during the rating of apps.

#### Performance and Efficiency

One of the most important factors contributing to the functionality of an app is its performance score, and how efficiently it can run and provide results on the user’s device. The argument is notched up by one point in importance due to the wide scale of customizability of performance components in mobile devices across various global mobile brands. Therefore, as the same foot measurement app may perform differently across different mobile devices with regards to CPU performance, total memory usage, total battery life impact, the possibility of device heating, and other device-specific features, this domain was included as a criterion for rating foot measurement apps, which are known to use significant processing power and complicated processing algorithms to output foot dimensions. To assess the memory usage and battery power consumption of an app, raters tracked the battery usage of apps from the app settings (Android) or auxiliary software (iOS) at intervals of 15 minutes during usage. Raters kept note of performance issues for apps, including (1) whether the app suffered frame drops, (2) the state of the app during any performance throttling, and (3) whether there were any noticeable changes in device temperature during usage.

#### Usability

The usability of an app refers to the quality of the app’s system that is used to achieve the goals of the app. Usability involves the fields of social and behavioral science, and is also linked with the science of design [25]. In prior studies involving human-computer interaction and user-centered design, poor usability and the lack of a proper user-oriented design have been identified as two of the major reasons for low user adoption rates of mobile health (mHealth) apps [26]. Usability testing of a foot scanning app is crucial in determining whether the app has sufficient quality to attract the attention of its target user groups. In today’s technology era, when engaging with a mobile app, the user’s attention is divided between interaction with the mobile app itself and interaction with the environment [27]. Navigation and ease of use are important measures of app usability since the in-app screen sequence leads the user through different views of the app to obtain the desired information [24]. In prior comparison studies involving comparative app usability testing, it was discovered that the usability of an app in field settings compared to that in laboratory settings varies greatly due to differences in user behavior and the user experience [27]. This signifies that usability testing of an app is a necessary stage in the app development cycle.

Taking into account prior work on app rating systems [13,18,19], we hypothesized that an app’s usability could be assessed as “good” given that (1) the app can be operated with ease; (2) the app’s navigation flow is uninterrupted; (3) the gestural design (if present in the app) and screen links (buttons, arrows, navigation panels, etc) are consistent across all app pages; and (4) the app provides an interactive experience by taking input from the users and giving feedback when necessary. These conditions were checked by the raters when rating the usability criteria of apps.

#### Measurement-Specific Functionality

In a foot measurement–specific app, the dimensionality of the features provided by the app is very important. In simple terms, if app A can measure more foot properties than app B, then the potential utility of app A can be considered to be higher than that of app B. Strategizing on this, we decided to include different types of apps that are directly or indirectly involved with the process of foot measurement in this analysis. We reviewed many studies on foot dimension measurements. Currently, foot dimension extraction heavily depends on 3D scanners that are used in both commercial and research areas specifically for measuring foot dimensions [20]. Other studies explored various techniques such as digital light project technology, image sensors, and 3D digitizing devices, including second-generation Kinect, to measure foot length, foot width, and metatarsal/ball girth [21-23]. However, footwear and insole design, instep and medial arch height, ball girth, and forefoot tilt are also necessary foot measurement dimensions [28-30]. In other works, laser scanners were used to scan foot length. The scanned data can also be refined and modified using laser scanners, and can be used for remodeling the human foot [31-33].

Taking these important pedorthic guidelines for foot measurement into account, the app measurement-specific functionality category considered for the weighing of foot measurement apps included the following measurement properties: (1) foot length, (2) foot width, (3) arch height, (4) instep girth, (5) joint girth, (6) short heel girth, (7) long heel girth, (8) heel width, (9) shoe size, and (10) forefoot tilt. Additional discoveries about taking inputs from camera sensors/images, the requirement of calibration markers or extra setup, along with the possibility of reconstruction of a 3D model of the foot were scoped into the rating scheme as functionality subscale items.

#### Transparency

Mobile apps that use social and personal information for their functioning are common targets for various businesses that capitalize on personalized services [34]. Apps frequently sell private information that is critical to an individual’s everyday livelihood without their awareness or approval, and the main cause of this is an improper mobile privacy policy. It must be ensured that when a user gives their consent to private data being accessed by apps, the apps strictly follow specific forms of data protection and regulation rules, and explicitly express to the users how and why their data are being collected, even if users may not understand the direct consequences of this action. Betzing et al [34] examined how increased transparency regarding personal data processing practices in mobile permission requests impacts users in making informed decisions, demonstrating that increasing the transparency of data processing practices increases users’ comprehension of their consent decisions. This suggests that obtaining user consent and following data protection rules are important items for the transparency of an app. In the case of foot measurement apps, the above-mentioned constraints should be followed along with verification of the publisher or developer as to whether the source of the app can be trusted and whether the app is successful in meeting its goals as described in the store description, which are also subject to scrutiny. With this information, a user can make an informed decision before downloading the app by determining the app’s authenticity.

To assess the transparency criteria of the selected apps, the following points were considered: (1) providing a general notice or an alert to users before accessing the user’s personal information, geolocation information, or private media files; (2) clearly specifying the intention of use of permissions by the app; (3) whether the app store page contained information and a description that aligned with the app goals; (4) whether the app conflicted with the user’s activities in unexpected ways; and (5) whether the techniques in the app are feasible to provide the desired output as claimed by the developer.

#### Subjective Quality

An app’s subjective quality refers to its users’ key views on the app. These can include personal app ratings, good and bad comments about the app, preference to pay for an app based on its features, and preference to recommend and use an app based on its relevance to the user. Often, users can deduce what an app offers by perusing the reviews from previous users. However, this is subjective, since the general distributed value of app comments and ratings saturate toward an approximated value only as the number of reviews on an app becomes large; thus, this approach to measuring the performance of an app predownload is not effective for apps with few or no user ratings/comments in the app store. However, users often make comparatively in-depth comments on apps with key points that are helpful during the app review phase, which is an optional but valid criterion for apps retaining the saturated direction from user reviews and comments.

We adopted a similar approach for the subjective quality assessment. The app raters answered questions about the degree of satisfaction of use, potential frequency of use in the future, overall app rating, and how likely they were to pay for the apps.

#### Perceived Impact of App on Users

When an app is downloaded by users, its impact becomes a notable indication of the potential usefulness of the app. Technological developments and ongoing breakthroughs in the fields of computer science and machines have helped thousands of users by guiding their health and preventing death, with a large number of apps designed to improve user health [35]. The main objectives of mHealth apps are to induce awareness about a particular health problem, and increase the user’s motivation to avoid and prevent future occurrences related to health. Additionally, mHealth apps may provide intervention techniques and advice useful in decreasing the user’s negligence toward their health and increasing help-seeking behaviors targeting solutions to health problems. However, Milne-Ives et al [36] found that most health-oriented apps yield little to no evidence of effectiveness in cases of patient health outcomes and health behavioral changes.

To conclusively support foot measurement apps as useful tools for changing the outcomes of foot health and attention-based behavior, the effectiveness of these apps must be evaluated by app users [36]. Toward this end, the following example strategy was used for assessing the utility of user comments for the rating of apps. The user comments were divided into two types based on the user’s review rating of the app: the comment was considered “good” if the rating was 4 stars or above, and otherwise was considered “bad.” For iOS apps, the number of downloads could not be viewed publicly and was thus excluded from influencing the assessment of apps with these criteria.

Understanding the impact of an app on users is not directly quantifiable. Therefore, in addition to the approach above, the following strategies were used to assess the extent to which an app was able to make an impact on its users based on their reviews and ratings: (1) how streamlined the app’s measurement process was, (2) the reaction of users about certain aspects of the app (store reviews), (3) whether the app had any outstanding features that appealed to the public regarding foot health and foot problems, and (4) whether the app contained information for raising public foot health awareness.

## Results

### Summary of Search Results

The initial searches using the primary and secondary search terms yielded a set of 145 apps across the two app stores, 41.4% (60/145) of which were excluded from any further review to satisfy the exclusion criteria of app duplication across stores. Of the remaining apps, 35.2% (51/145) were excluded due to failing to satisfy the inclusion criteria of relevance to foot measurement and not having any app store rating or comment. Of the remainder, after installing and using the apps, 5.5% (8/145) were excluded due to various app module errors and store restrictions. In total, 26 apps were selected as eligible to be reviewed as foot measuring apps using our proposed modified rating scale.

### Overall Assessment of the Apps

The overall assessment of all eligible apps in this study (reported in Table 2) led to the discovery that most apps belong to the 2D and 3D scanning subcategories. The categorical distribution of all the reviewed apps is shown in Table 3.

**Table 2:** Assessment scores for foot measurement apps.

App name	Aesthetics	General	Performance	Usability	Functionality	Subjective	Transparency	Impact	Mean (SD)
ShapeCrunch	4.00	3.29	4.33	4.00	2.33	3.75	4.67	4.40	3.85 (.74)
INESCOP YourFeet	5.00	3.00	4.67	4.75	1.73	3.25	5.00	2.60	3.75 (1.26)
FISCHER Scan-Fit	5.00	3.00	4.17	4.00	2.45	3.75	4.67	3.00	3.75 (.88)
Foot Measure	2.50	1.50	3.83	3.75	1.73	1.00	1.33	1.00	2.08 (1.16)
SizeMyShoe	4.25	3.00	4.67	4.00	1.36	2.25	3.67	2.40	3.20 (1.14)
ATLAS-scan your feet!	4.25	3.00	3.33	4.25	1.36	4.00	5.00	2.20	3.42 (1.2)
Jenzy: Easy Kid Shoe Sizing	5.00	3.29	4.67	5.00	1.36	4.25	5.00	2.00	3.82 (1.45)
Shoe Size Meter-foot length	4.25	3.00	4.67	3.75	1.36	3.50	4.33	3.60	3.56 (1.03)
Shoe Size Converter	4.50	1.57	5.00	4.50	1.36	3.50	4.67	2.80	3.49 (1.43)
The Foot Fit Calculator(BikeFit)	5.00	2.14	4.50	4.50	1.67	3.00	4.00	3.60	3.55 (1.19)
Foot Length Converter Size in Lite	4.00	1.5	5.00	4.00	1.36	2.50	4.33	2.20	3.11 (1.39)
myFoot-Rescue your feet!	4.25	4.00	4.67	4.75	3.00	4.00	4.75	4.40	4.23 (.58)
Remeasure Men Body	4.75	4.00	5.00	4.50	1.00	4.75	5.00	3.20	4.03 (1.36)
FootFact	3.25	2.00	4.67	4.25	1.67	1.50	2.33	1.60	2.66 (1.25)
Swift Orthotics	4.50	1.50	4.00	4.25	2.09	4.00	3.00	3.20	3.32 (1.08)
Nimco Professional Shoe Sizing	5.00	4.20	4.50	4.50	4.33	4.50	4.67	3.40	4.39 (0.46)
Shoe-buddy	4.25	3.67	4.17	4.00	2.33	4.00	4.75	3.60	3.85 (0.71)
3D Avatar Feet	4.25	2.14	3.67	4.25	2.67	4.25	4.75	3.20	3.65 (0.91)
SUNfeet	3.75	2.71	4.00	3.75	2.67	3.75	5.00	2.80	3.55 (0.80)
ECLO	5.00	3.86	4.33	4.75	2.33	4.00	5.00	2.40	3.96 (1.07)
FotAppenScan	1.50	1.57	4.50	2.50	1.67	2.25	3.00	2.20	2.40 (0.99)
Ortholutions	4.75	1.50	4.33	4.50	1.67	4.25	5.00	4.20	3.78 (1.38)
AARA Orthotics	4.50	3.50	4.00	4.25	1.67	4.00	4.75	4.40	3.88 (0.97)
Aqualeg	1.75	2.00	3.67	1.50	1.67	2.25	2.00	1.20	2.01 (0.75)
Anodyne Scanner	4.50	2.00	4.00	4.50	1.67	4.25	4.75	3.40	3.63 (1.19)
3DsizeMe	4.75	3.00	4.33	5.00	1.67	4.50	4.75	3.40	3.93 (1.15)

**Table 3:** Categorical distributions of the reviewed foot measurement apps (N=26).

Subcategory	Count, n (%)
3D Foot Scanner	9 (35)
2D Foot Scanner	8 (31)
Shoe Recommender	4 (15)
Simple Size-unit Converter	2 (8)
Foot Progress Tracker	2 (8)
Foot Tilt Calculator	1 (4)

The 2D and 3D scanning subcategories are the most important since they handle output as raw measurement values, which can be used to make custom-made shoes or to recommend an individualized shoe fit, and to provide users with foot dimension measurements that may further be used to maintain foot health and prevent foot-related problems. Apps in these two categories mostly use calibration markers such as standard-sized papers (eg, A4, A5) and purchasable barcode stickers. They use the camera sensors on mobile devices to capture, process, and measure users’ foot dimensions. Of these apps, 31% (8/26) are the 2D scanning type and 35% (9/26) are the 3D scanning type. Seven of the nine 3D scanning apps require an external sensor (eg, Structure sensor, KinectV2) for the app to function properly and all of these apps can reconstruct a 3D foot model, which is valuable since 3D foot model data are useful for making custom shoes.

Most of the apps targeted a general group of users with no age requirements whereas the apps specifically categorized as 2D and 3D foot scanning apps (17/26, 36%) targeted young adults and adolescents. There were two apps, Jenzy: Easy Kid Shoe Sizing and Remeasure Body, which targeted users in younger age groups (2-18 years old). However, the Remeasure Body app was not a 2D or 3D scanning app but rather a foot progress tracking app. The assessment revealed many commercial apps that use advanced image scanning and processing techniques to determine foot size, shoe type, and size, and even reconstructed 3D foot models to obtain shoe and insole sizes (ATLAS-Scan your feet!, ECLO, Fischer Scan-Fit, Nimco Professional Shoe Sizing, Jenzy: Easy Kid Shoe Sizing, ShapeCrunch).

The assessment results showed that the overall navigability, design, and appeal of current apps scored higher than other aspects of the apps, with usability ranging from 3.75 to 4.50 (Table 2). However, the general range of the measurement-specific functionality was low for all of the apps reviewed, except for Nimco Professional Shoe Sizing. This app performed better in terms of its ability to measure foot length and width, foot instep height, and ball girth. The app also includes a 3D model reconstruction feature of the foot using the procured measurement values. An additional feature of the app is that it can suggest shoe shape and size based on the processed 3D foot model.

Except for one app, all others on the list were free to download, with 31% (8/26) of the apps having subscription packages available; these were essential to obtain full access to the apps’ functionality suite (limited access is free).

The mean overall app rating was 3.49 out of 5 (Table 4). Significant differences were detected across domains, most notably impact, general features, and functionality received the lowest ratings. In contrast, the most highly rated domains were performance and transparency. Other domains that scored higher mean values were aesthetics and usability (Table 4).

**Table 4:** Overall app ratings.

Assessment criteria	Assessment score	
	Mean (SD)	95% CI
Aesthetics	4.17 (0.95)	3.79-4.56
General	2.69 (0.90)	2.33-3.05
Performance	4.33 (0.44)	4.16-4.51
Usability	4.14 (0.74)	3.85-4.44
Functionality	1.93 (0.70)	1.65-2.21
Subjective	3.50 (0.97)	3.11-3.89
Transparency	4.24 (1.04)	3.82-4.66
Impact	2.94 (0.94)	2.56-3.32
Total	3.49 (0.61)	3.25-3.74

### Internal Consistency of the Modified Scale and Interrater Reliability

We used Cronbach α [37] to calculate the internal consistencies of the overarching domains of the modified rating scale: aesthetics, general app features, performance and efficiency, usability, measurement-specific functionality, transparency, subjective quality, and perceived impacts on users. For this work, one of the foot measurement apps reviewed in this study, INESCOP YourFeet, was additionally used to rate the internal consistency of our modified rating scale. All three independent raters considered the internal consistency of all subscale items as “good” to “high.” The overall internal consistency of the modified rating scale was high at α=.84, which is considered excellent (eg, [38]). The Cronbach α values were also in the range of good to excellent for the other subscales: aesthetics (α=.88), general features (α=.92), performance (α=.70), usability (α=.84), functionality (α=.92), subjective (α=.79), transparency (α=.90), and perceived impacts (α=.78).

The same method was used to measure the interrater reliability of our raters who independently reviewed the set of 26 apps in this study. The interrater agreement score ranged between .54 and .70, which is considered as a fair to good level of rater reliability or agreement [38].

### Comparison of Store Ratings and Rating Scales

The store ratings of the reviewed apps were compared to the score of the apps from our rating scale (Table 5). The standard deviation of the difference in the two scores for the reviewed apps was 1.07. This deviation is not too poor considering that the score in our rating scale is an aggregated mean of the various domains that are necessary to specify the quality and criteria of foot measuring apps. Even though the ratings were within a close range of spread, there is a need for more than just the store rating to motivate users to install and use the apps since the apps reviewed in this study are not intended to be used as casual apps (for evaluation of the study’s objective). During the review, no star ratings for the apps SUNFeet, Ortholutions, AARA Orthotics, and Aqualeg were found, and thus these apps were excluded from the calculation of the total standard deviation.

**Table 5:** Comparison of app ratings from the app stores and the developed rating scale.

App name	Measured score	App store rating
ShapeCrunch	3.85	4.50
INESCOP YourFeet	3.75	4.10
FISCHER Scan-Fit	3.76	2.10
Foot Measure	2.08	2.00
SizeMyShoe	3.20	2.10
ATLAS-scan your feet!	3.42	2.00
Jenzy: Easy Kid Shoe Sizing	3.82	3.30
Shoe Size Meter-foot length	3.56	3.70
Shoe Size Converter	3.49	4.90
The Foot Fit Calculator (BikeFit)	3.55	3.00
Foot Length Converter Size in Lite	3.11	3.00
myFoot-Rescue your feet!	4.23	3.40
Remeasure Men Body	4.03	4.40
FootFact	2.66	3.10
Swift Orthotics	3.32	5.00
Nimco Professional Shoe Sizing	4.39	2.00
Shoe-buddy	3.85	3.30
3D Avatar Feet	3.65	4.50
ECLO	3.96	5.00
FotAppenScan	2.40	3.50
Anodyne Scanner	3.63	5.00
3DsizeMe	3.93	4.20

### Measurement Criteria for Apps

The key function of foot measurement apps is the measurement of foot dimensions so that the users are aware of the current condition of their feet. In accordance with the selected measurement criteria for foot measurement apps, we evaluated the comprehensiveness of the reviewed apps regarding the criteria. As shown in Table 6, there was substantial variation in the number of foot dimensions measured by the apps. Table 6 provides a transformed view of the number of measurement items of apps as discussed in the Measurement-Specific Functionality subsection of the Methods section.

**Table 6:** Assessment criteria for the measurement functionality of apps.

Measurement criteria	Google Play Store (n=11), n (%)	Apple App Store (n=15), n (%)	Total (N=26), n (%)
Foot length	6 (55)	7 (47)	13 (50)
Foot width	6 (55)	5 (33)	11 (42)
Foot arch (medial) height	1 (9)	2 (13)	3 (12)
Foot instep girth	0 (0)	1 (7)	1 (4)
Foot joint girth (ball girth)	0 (0)	3 (20)	3 (12)
Short heel girth	0 (0)	0 (0)	0 (0)
Long heel girth	0 (0)	0 (0)	0 (0)
Heel width	0 (0)	2 (13)	2 (8)
Shoe size	2 (18)	2 (13)	4 (15)
Forefoot tilt	0 (0)	2 (13)	2 (8)
3D foot model reconstruction	1 (9)	10 (67)	11 (42)

The general distribution of the measured foot dimensions of the reviewed apps was very low overall. From the total of 26 apps, only 50% can measure foot length, 42% can measure foot width, 15% can determine shoe size for users, 12% can measure foot instep height and ball girth, and 4% can measure foot instep girth and heel width. Important dimensions that are crucial for the construction of shoes, insoles, and foot wedges (eg, for bicycle pedals, saddles) were missing from the measurement features offered by all of the reviewed apps. Most of the reviewed apps from Google Play Store were poor compared to those available from Apple App Store. However, since apps were excluded based on duplication across app stores and region-restriction criteria, this information should not be used to reflect the current state of platform-specific apps (ie, Android devices have a lower variety of apps devised for foot measurement compared to iOS devices).

Further evaluation of the measurement criteria showed that the maximum number of dimensions measured by a foot measuring app was five. No app measured more than half of the required foot measurement criteria, and half of the apps (50%, 13/26) only measured one dimension. There were 3 (12%) apps that measured five dimensions, 3 (12%) apps that measured four dimensions, 1 (4%) app that measured three dimensions, 3 (12%) apps that measured two dimensions, and 3 (12%) apps that measured no dimensions at all. In general, most of the reviewed apps were not suitable for foot measurement in clinical practice for custom-made shoes and insoles/orthoses or for general use by the public.

### User Reviews from App Store

User reviews provide a rich source of information about the performance and future viability of apps. Hence, to drive the commercial success of apps (in any category), developers and publishers aim to receive good and positive reviews as these are a crowdsourced quality indicator of apps [39].

The analysis of user comments regarding the foot measurement apps from both stores showed that most of the apps lack good feedback or positive reviews from app users, since the number of downloads of the related apps was generally low and an estimated 31% (8/26) of the apps had only 100 to 1000 users. The number of downloads of iOS apps could not be viewed and there were too few reviews with information from users for proper assessment of these apps. These findings are similar to those of Vasa et al [39], who indicated that users tend to write little to nil when reviewing an app that performs better in its category. We found that 42% (11/26) of the apps had very short or no informative reviews to support their ratings.

However, 54% (14/26) of the apps had some informative reviews in the app stores, which were contrasted with their ratings/scores. One of the most positive sets of user responses was found for the app ShapeCrunch. This app is a foot scanner that uses machine learning–generated 3D printed insoles to reduce the discomfort of foot pain. Based on the reviews of several users, this app is rated excellent on the grounds that it can provide properly fitted insoles for users with a wide variety of foot problems. The app Swift Orthotics also received a good review, which was consistent with the overall ratings both in the app store and in the modified evaluation scale. This app uses augmented reality technology to measure foot dimensions, and was the only other app that could measure heel width along with Fischer Scan-Fit among the reviewed apps. Strong positive feedback with no mention of drawbacks was received for the apps ECLO and 3DSizeMe, which were positively correlated with the ratings of the independent raters, although ECLO is a shoe recommendation app that can order shoes based on user scans from the ECLO online store. The app 3DSizeME has great support for Structure Sensor, an advanced 3D scanner, and there are many companies that can directly support 3DSizeMe file formats to order custom shoes and insoles. By contrast, apps such as Aqualeg, Ortholutions, and AARA Orthotics, which use external scanning mechanisms, did not receive much public exposure although they scored high ratings according to the devised rating scale, and all of these apps had features for visualizing and either uploading foot scans to company servers or exporting the model data to order shoes, insoles, and even medical casts for different body parts. Similar results were found for the 2D scanning app INESCOP YourFeet, which did not have good store descriptions, but the store ratings were consistent with our measured ratings. However, this app lacked good functionality in terms of providing detailed foot information to make custom footwear (although it can measure regular shoe size).

Our raters found inconsistencies between app user reviews and the actual performance of some apps: Shoe Size Meter-foot length, SizeMyShoe, FISCHER Scan-Fit, Jenzy: Easy Kid Shoe Sizing, INESCOP YourFeet, and The FootFit Calculator. Two of the apps that performed better than the rest of the 3D scanner apps reviewed were SUNFeet and 3D Avatar Feet; however, their respective app store pages had no user reviews at all.

From the above analysis of foot measurement app user reviews, we highlight the inconsistency of rater app ratings in this study and the app store user reviews. This may be because the performance and functionality of apps are dependent on the specific device version and software being used. This suggests that most of the currently available apps suffer from device architecture and build-related problems, and need to be further optimized. Another possible cause of this rating versus review inconsistency is that when users are rating apps on the store, they generally do not focus on domains such as perceived impacts, transparency, and the technical functionality of the apps, and thus their comments are not representative of all of the apps’ features.

## Discussion

### Principal Findings

The findings of this review are discussed from three main aspects: (1) the viability of apps in podiatric practice for making custom shoes, (2) the viability of apps for individual use for general measurement purposes, and (3) the potential of inducing behavioral changes about foot health and foot-related problems among users.

This review demonstrates that although there are a handful of foot measurement apps available in commercial stores, the performance of the apps with regard to the objective(s) of this study is poor and the features are insufficient. The objective of foot measuring apps available in app stores to be used in clinical practice to facilitate custom-made shoes is not being achieved using the current configuration. Although apps may be used for different purposes such as online shopping for shoes and insoles, and the casual measurement of feet, they are deemed unusable as pedorthic tools for the professional measurement of foot dimensions, which require comprehensive fulfillment of the measurement criteria determined by this study to achieve proper and precise measurements for custom-made shoes and insoles.

Most of the reviewed apps met less than half of the measurement dimension criteria required to properly measure the feet of users. Some of the apps that were used for foot measurements did not provide sufficient relevant information about the actual foot dimensions they measured; rather, this information was used and processed for other purposes. No app included any information about the degree of accuracy that could be achieved from the measurement with the used technologies.

In general, most of the current apps belonging to 3D categories could reconstruct a 3D model of the feet. Some apps measure a small number of foot properties and send scanned information to their internal servers to construct a 3D foot model. Other apps take in information to find and fit custom-made shoes and insoles/orthoses for users (possibly with foot-related disabilities and zonal pain), and have them delivered to the user’s home. However, except for 6 (23%) apps (Nimco Professional Shoe Sizing, Fischer Scan-Fit, 3D Avatar Feet, SUNFeet, 3DSizeME, and Anodyne Scanner), no other app reported the user’s foot dimension information with sufficient detail to enable custom-made shoes (and insoles) to be built. The app 3DSizeME is mentionable in this respect owing to its performance and data shareability options, which are lacking in most current apps, thus making the mobility of data difficult. In general, the apps tended to focus on particular measurement criteria, which cannot completely describe the structure of the foot to make custom-made shoes.

With respect to the general measurement use of individuals, most of the reviewed apps were either developed for commercial purposes or were most likely to be used as lookup apps to obtain preferred foot/shoe sizes. However, the usage frequency of apps underperforms by a large margin; 65% (17/26) of the apps subjectively reviewed by raters had an average subjective usage frequency of under 10 times over 12 months, 31% (8/26) of the apps had a usage frequency of 10 to 50, and only one app was properly usable with a usage frequency ≥50 over 12 months. Apps in the shopping category that met this usage range were ATLAS-Scan your feet!, Shoe Size Converter, and Nimco Professional Shoe Sizing. The usage statistics and the calculated ratings of such apps in this review, together with analysis of the user reviews from the app stores, suggest that the cause of the poor usage of apps, even though a large percentage of these apps provided all of the included features for free, was a lack of more features and the presence of poorly optimized features in the apps. Thus, for individual use, the apps are not likely to be used as foot measurement tools of high value. Some apps had detailed complaints about their inability to properly size feet and shoes, while others suffered from performance issues, including battery draining, network errors, and overheating problems with prolonged use. In some apps, the structural flow of technical aspects was confusing and difficult for the users to follow.

This review showed that the apps that fell into the shopping category performed better in calculating foot measurements compared with the foot-scanning apps that output raw measurement values. Seven out of 26 apps met this criterion, and all apps had consistently good mean scores.

Based on the accuracy of their store descriptions and the credibility of the app developers, the apps were mostly consistent about their intended use; however, some apps were not explicit about the consent to the use of data. Some apps displayed warning dialogs to notify users about providing private data access, whereas some apps did not offer sufficient information about their policy on protecting the personal and private information of users on their support websites or on the app privacy policy page (if relevant), which raises a question about their intention to ensure the privacy of user data.

The results also showed that current apps have low fidelity for inducing behavioral changes to promote foot health and an awareness of foot-related problems. The apps mostly did not meet the required basic measurement dimensions needed to properly measure feet, and were therefore deemed to be unsuitable for inducing health-related awareness (79.3% of app responses were negative) and help-seeking behavior (82% of app responses were negative) related to foot health and problems. However, this study showed that most of the apps used in cases other than the direct measurement of foot dimensions had a positive effect (64.7% positive responses) on the necessity of buying properly sized shoes and insoles. Such impacts suggest that although the commercial market has grown with the increase in foot measurement–related apps, the technical quality of the apps needs further improvement. Developers should carefully follow guidelines such as those provided by our rating scheme when developing apps that are publicly available for making custom-made footwear or individualized shoe fit for all types of feet.

### Strengths and Limitations

The search methods that were used in this study followed a modeling pattern similar to that of previous studies involving various mHealth categories, including the management of various mental and chronic diseases [26], diet [10], drugs and alcohol [40], physical activity [36], weight [41], and diabetes [11,12]. This work focused on apps that were not access-restricted by region. Various domains of software characteristics hypothesized to be important for foot measurement were presented and a rating scale was created based on thorough reviews of published foot measurement apps. Although these apps were not verified for the presence of applied knowledge of foot morphology and foot health, a general impact value of the apps was taken to assess how the consumers viewed the features provided by these apps and whether they could produce a sense of awareness about foot health. It should be noted that despite raters independently testing all of the selected and eligible apps for the review, these values should not be interpreted to focus on any particular criteria or item, as this was not the main objective of the study. The findings presented are a broad characterization of the general quality characteristics and measurement-specific features that should be presented in foot measurement apps to create custom footwear products and for general use by individuals to enhance awareness of foot health, and thus represent the current state of the commercial app market with regard to foot measurement apps. This study provides insight into the technical knowledge of developers and publishers about foot health and foot measurement, and points to possible directions to advance research and development in the foot health and measurement categories.

### Future Directions

Although we aimed to assess all of the important and relevant software characteristics of apps, certain assessment criteria such as the accuracy of the measured dimensions of feet, CPU, memory, and battery usage could not be assessed accurately due to resource constraints. Hence, future research will focus on determining foot measurement accuracy and introduce more app stores for an even more comprehensive review. Future work will also investigate the possibility of including more features as part of the categorical criteria to ensure more robust foot measuring apps with enhanced technical features. Another focus of this research would be a direct extension and update of the review since, among the vast collection of foot measurement apps reviewed for this study, we may have missed apps because of our search criteria, and there may be apps that were not available due to the regional restriction of the app stores.

### Conclusion

The conclusion drawn from this review on foot measurement apps is that most mobile apps in the app stores do not sufficiently meet the criteria required to manufacture custom-made footwear or recommend an individualized shoe fit. Although a few apps provide some information about the user’s feet, they require users to enter the data manually. We believe that only by addressing the entirety of issues found and by applying more caution to implementing features that are required for the completeness of foot measurement will developers be able to bring useful apps to app stores that are suitable for direct professional use in clinical practice for custom-made footwear. Such apps will also motivate individual users to properly address foot problems and become more aware of the importance of foot health.

## Abbreviations

MARS: Mobile App Rating Scale
mHealth: mobile health
